# Currarino's syndrome misinterpreted as Hirschsprung's disease for 17 years: a case report

**DOI:** 10.1186/1757-1626-2-118

**Published:** 2009-02-03

**Authors:** Hooshang Saberi, Zohreh Habibi, Amin Adhami

**Affiliations:** 1 Department of Neurosurgery, Imam Khomeini Hospital, Tehran University of Medical Sciences, Tehran, Iran; 2 Brain and Spinal Injury Repair Research Center, Tehran University of Medical Sciences, Tehran, Iran; 3 Department of General Surgery, Imam Khomeini Hospital, Tehran University of Medical Sciences, Tehran, Iran

## Abstract

**Background:**

Currarino's syndrome is an autosomal dominant hereditary disease known by the triad of anorectal stenosis, anterior sacral defect, and a presacral mass that is most often an anterior sacral meningocele. Actually this syndrome could remain asymptomatic in many instances, but symptomatic patients might present with constipation as the sole manifestation of the Currarino's syndrome among the other wide spectrum of manifestations.

**Case presentation:**

An 18-year old woman was diagnosed with a late-recognized Currarino syndrome, presented by a longstanding constipation which had been wrongly diagnosed and treated as Hirschsprung's disease since early childhood.

**Conclusion:**

Long-lasting constipation could imply to neural tube anomaly such as anterior sacral meningocele with or without association to Currarino's syndrome.

## Background

Currarino's syndrome is an autosomal dominant hereditary disease known by a triad of anorectal stenosis, anterior sacral defect, and a presacral mass that is most often an anterior sacral meningocele (ASM) [1]. Although may remain asymptomatic, the lesion can considerably achieve a large size and accordingly exerts mass effect on the pelvic structures leading to the manifestations like constipation, rectal fullness, lower abdominal pain and dysuria [2]. Therefore, the clinical condition could be erroneously appointed to the problems with gastrointestinal origin.

In this report an 18-year old woman with anterior sacral meningocele and other signs compatible to the triad of Currarino's syndrome, was presented with a longstanding constipation which had been wrongly diagnosed and treated as Hirschsprung's disease since early childhood.

## Case report

An 18 year-old lady was admitted because of failure of stool passage for the last 2 months. She had experienced several episodes of partial bowel obstruction within the last years likewise. The patient had been coined to have Hirschsprung's disease after doing barium enema and colonoscopy since she was one year old. However, rectal biopsy was not performed for pathologic confirmation of the disease due to her parents' refusal. The patient was treated by various laxatives for constipation without any significant improvement with her defecation problem. There was no history of genitourinary problems or phacomatosis. None of the parents or her siblings had history of anorectal disease likewise.

Proctology examination revealed a ventrally located anus, and a round well-circumscribed compressible mass in retrorectal area. There was no evidence of saddle hyposthesia or sphincter problem in neurology examination.

**Figure 1:**
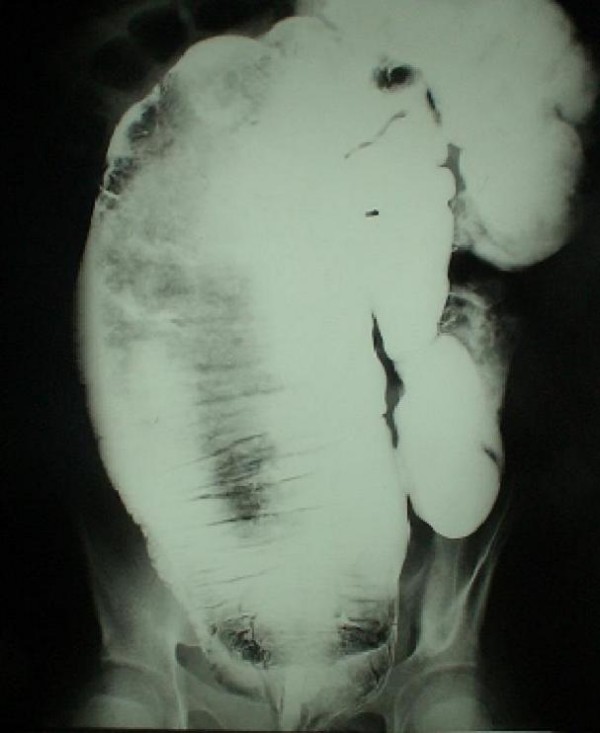
**Barium enema depicting dolichoectatic colon and effacement of normal haustrations**.

**Figure 2:**
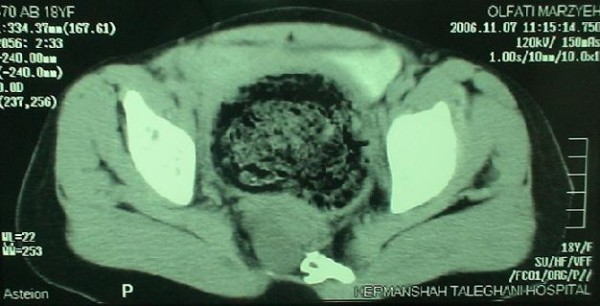
**Axial pelvic computed tomography depicting erosion and scalloping of the right hemisacrum by the retrorectal mass**.

Barium enema demonstrated an ectatic descending colon, with less prominent colonic haustrations. The ectatic segment tapered to a narrow ventrally displaced rectum (fig 1). In Plain pelvic radiography a scimitar sacral defect could be seen, and computed tomography delineated scalloping of the right hemisacrum caused by a round homogenous hypodense presacral mass (fig 2).

The patient planned for elective en-block resection of the mass. A midline posterior incision was made between the coccyx and the ventrally located anus but surprisingly a clear liquid resembling cerebrospinal fluid gushed out from the incision. Accordingly, the wound was closed, and the operation canceled for further evaluation and assessment.

**Figure 3:**
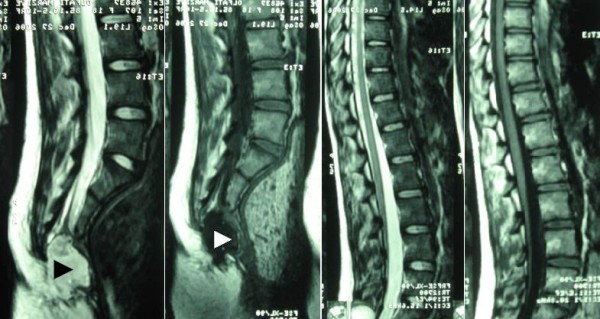
**Sagittal magnetic resonance images demonstrating anterior sacral meningocele originating from the caudal thecal sac (arrowheads), associated with thick tethered filum terminale**.

Following a neurosurgery consultation, magnetic resonance imaging (MRI) was performed one day after surgery which revealed an anterior sacral meningocele originated from the caudal thecal sac. The lesion had displaced rectum and anus to anterior. The tip of the conus medullaris was ended at the level of S1, connected to the thick tethered filum terminale (fig 3).

During the planned surgery in the following week a sacral laminectoy was performed to obliterate the meningocle by ligating the neck of the lesion, and L5 laminectomy was done to release the tethered cord then after. The patient's bowel habits successfully returned to normal soon after the operation. The presacral mass disappeared expectedly, and the patient remained symptom-free after 1 year follow-up.

## Discussion

Anterior sacral meningocele was first described in 1837 as a part of neural tube defect (NTD) spectrum [3]. The lesion results from a hernia through a defect on the anterior surface of the sacrum. The outer wall of the herniated protrusion is a dural membrane and the internal one is an arachnoid membrane, originating from the ventral aspect of the thecal sac [3].

Sacral malformation, presacral mass, and anorectal malformations comprise the classic Currarino's triad [4,5]. The presacral mass in this triad could be an ASM, a teratoma, or an enterogenous cyst, with the leading cause being ASM [3]. Other associated symptoms and malformations include neonatal-onset bowel obstruction, chronic constipation, recurrent perianal sepsis, urinary tract anomalies, female internal genital anomalies, and tethered spinal cord [5]. Currarino and associates represented the previously described anomalies as a syndrome in 1981 [6].

The Currarino's triad is an autosomal dominant disorder linked to the 7q36 locus [4]. About 50% to 60% of the reported cases have a family history of triad-associated anomalies [1,2]. The condition is recently proposed to be associated to the mutations of the HLXB9 gene which encode the HB9 transcription factor and interact with DNA early in embryological development [4].

Although Currarino's syndrome accounts for an uncommon cause of constipation [1], prolonged constipation may be the only presentation of many patients. Hence, long-lasting constipation should alert the physician as a possible and sole presentation of this syndrome in suspicious cases. The pathogenesis may be attributed to; [1] mechanical compression, [2] tethered spinal cord, [3] sacral nerve roots compression, and [4] anterior located anus. In this patient, rapid resumption of normal bowel habit could be an explanation in favor of the mechanical compression hypothesis.

Magnetic resonance imaging is a sensitive and specific noninvasive diagnostic modality, and could be performed in any patient with long-standing constipation suspicious to have concurrent neural tube and anorectal anomalies.

## Conclusion

Long-lasting constipation could imply to neural tube anomaly such as anterior sacral meningocele with or without association to Currarino's syndrome. Therefore, it might be beneficial to carry out MRI in otherwise healthy patients with prolonged constipation when a definite cause could not be confirmed straightforward.

## Competing interests

The authors declare that they have no competing interests.

## Authors' contributions

HS: performed the Neurosurgery and gave the final approval for the version to be submitted. ZH: made contribution in conception and collecting data and drafting the manuscript. AA: was the general surgeon counselor and contributor in revising the manuscript critically.

## Consent

Written informed consent was obtained from the patient and his parents to publish this case report and accompanying images in "Cases journal". A copy of the written consent is available for review by the Editor-in-Chief of this journal.
